# Photodynamic therapy of lung cancer, where are we?

**DOI:** 10.3389/fphar.2022.932098

**Published:** 2022-08-30

**Authors:** Anine Crous, Heidi Abrahamse

**Affiliations:** Laser Research Centre, Faculty of Health Sciences, University of Johannesburg, Johannesburg, South Africa

**Keywords:** photodynamic therapy, lung cancer, nanomedicine, photosensitizer, nanoPDT, nanomaterials, nanotechnology

## Abstract

Lung cancer remains the leading threat of death globally, killing more people than colon, breast, and prostate cancers combined. Novel lung cancer treatments are being researched because of the ineffectiveness of conventional cancer treatments and the failure of remission. Photodynamic therapy (PDT), a cancer treatment method that is still underutilized, is a sophisticated cancer treatment that shows selective destruction of malignant cells via reactive oxygen species production. PDT has been extensively studied *in vitro* and clinically. Various PDT strategies have been shown to be effective in the treatment of lung cancer. PDT has been shown in clinical trials to considerably enhance the quality of life and survival in individuals with incurable malignancies. Furthermore, PDT, in conjunction with the use of nanoparticles, is currently being researched for use as an effective cancer treatment, with promising results. PDT and the new avenue of nanoPDT, which are novel treatment options for lung cancer with such promising results, should be tested in clinical trials to determine their efficacy and side effects. In this review, we examine the status and future potentials of nanoPDT in lung cancer treatment.

## 1 Introduction

Cancer is still a major public health concern because of the high death rate in various parts of the world. Regardless of current therapies, there is a critical need for the development of novel therapeutic techniques to manage the condition across diverse healthcare systems. Cancer is caused by abnormal cell growth and regulation, which results in abnormal proliferation, neoplasia, and the ability to spread. The foremost cause of cancer-related death worldwide is lung cancer (Kordiak et al., 2022). When patients present to the hospital, they are typically diagnosed with advanced disease (Guerrini et al., 2022) and therapy resistance and relapse (Zhang et al., 2020). Early detection and treatment advances are expected to decrease lung cancer mortality.

Current lung cancer treatments are staged according to the International Association for the Study of Lung Cancer’s eighth edition staging system. The availability of positron emission tomography with computed tomography scanning and endobronchial ultrasound (US) for mediastinal lymph node sampling has improved the accuracy of lung cancer staging (Jones and Baldwin, 2018).

In the last few years, early-stage nonsmall cell lung cancer (NSCLC) surgery has made rapid improvements. A lobectomy is considered the gold standard of care for healthy individuals with early-stage lung cancer (Hartwig and D'Amico, 2010). Modern surgical techniques, such as minimally invasive video-assisted thoracoscopic surgery (VATS) for lung resections, are enabling better prognosis for patients and better operation conditions for surgeons, redefining surgical fitness (Jiang et al., 2020). In some studies, perioperative mortality and long-term survival following VATS lobectomy were found to be superior to those following open surgery (Jones and Baldwin, 2018). Because of the limited maneuverability and stereoscopy of standard VATS, it is not suitable for complex cases. Robot-assisted thoracic surgery (RATS) may overcome the limitations of traditional VATS. The RATS system is comprised of a remote console and three or four robotic arms capable of simulating surgeon movements. In addition, it provides surgeons with a magnified three-dimensional (3D) and high-definition operation field, which enables them to perform more complex procedures (Jiang et al., 2020). However, because of the novel surgical technique, the cost is higher.

Radiotherapy is used curatively and palliatively to treat lung cancer. Radiotherapy is recommended for people with early-stage NSCLC who cannot be operated on because of their health or the risks of undergoing surgery. Stereotactic ablative radiotherapy (SABR) is now known as a treatment option with a high chance of being a curative therapy (Baker et al., 2016). SABR can deliver large doses of radiation with precision by utilizing an external three-dimensional coordinated system that is linked to respiratory cycle movements. There are still uncertainties about the best radiotherapy dose fractionation regimen, and because of the high ablative doses used in SABR, there is a risk of peripheral organ toxicities. Certain scenarios, such as the treatment of central or recurring tumors, necessitate greater caution (Chia and Master, 2018).

Chemotherapy is a systemic treatment option for lung cancer and the standard for first- and second-line management of small cell lung cancer (SCLC) (Yang et al., 2019a). SCLC is comprised of pulmonary neuroendocrine cells that are epithelial in origin (Park et al., 2011) and has the tendency to metastasize and grow rapidly (Ko et al., 2021). It represents up to 15% of lung cancer cases worldwide, with a poor prognostic outcome of less than 10% of people having a 5 year survival rate (Torres-Durán et al., 2021). NSCLC is phenotypically and histologically different from SCLC where it consists of two major histological subtypes, namely, adenocarcinoma and squamous cell carcinoma. NSCLC is a slower-growing cancer for which the overall 5 year survival rate is 63% and decreases depending on the stage of the disease (Blandin Knight et al., 2017). SCLC has initial treatment sensitivity to chemotherapy but has been shown to recur along with metastasis (Ito et al., 2017). Chemo has been revolutionized for NSCLC through cancer cell targeting and has been tailored to the individual using driver genetic mutations (Jones and Baldwin, 2018). Targeted chemotherapy requires specific histological diagnoses and has been shown to be well tolerated by patients, with studies showing a longer progression-free survival compared with standard chemotherapy (Jones and Baldwin, 2018); nevertheless, chemotherapy still presents with severe side effects to the host and their immune system (Schirrmacher, 2019).

Immune checkpoint inhibitors, which work by inhibiting the programmed death ligand 1 and 2 receptors, are a relatively new class of systemic treatments (PD-L1 and PD-L2). These immunotherapies prevent cancer cells from evading immune detection by blocking the PD-L1/2 and PD-1 receptor pathways, allowing cancer cells to be identified and killed by cytotoxic T-cells. Patients with previously treated NSCLC who received this treatment instead of standard chemotherapy had a higher overall survival rate (Borghaei et al., 2015; Herbst et al., 2016). Likewise, immunotherapy has been shown to increase overall patient survival with SCLC (Yang et al., 2019a). These treatments are expensive, and their efficacy is modest. Despite their significant cost and lack of predictive biomarkers for patient selection, pembrolizumab and nivolumab have been approved by the National Institute for Health and Care Excellence for use within the cancer drugs fund, as first-line treatment for PDL-1 positive (>50% tumor staining) advanced-stage NSCLC (Jones and Baldwin, 2018), and nivolumab was approved by the Food and Drug Administration (FDA) for recurrent SCLC (Yang et al., 2019a).

More effective and alternative treatments for lung cancer patients are urgently needed. Because of their nonspecificity and cytotoxicity toward normal cells, patients succumbing to relapse, and immunotherapy relying on the patient’s immune system, the aforementioned treatments carry a high risk of systemic adverse effects. Photodynamic therapy (PDT) is an underutilized form of lung cancer treatment that has been extensively studied. PDT has been shown to be an effective cancer therapy strategy in numerous clinical studies and showed to improve quality of life and survival in inoperable cancer patients (Wang et al., 2021). Although PDT has advantages such as reduced drug resistance and minimal dark toxicity, the lipophilic character of most photosensitizers (PSs), the narrow half-life of PS in plasma, poor tissue infiltration, and moderate tumor specificity still limit its use in clinical practice. To overcome PDT limitations, nanocarriers have been incorporated and studied, aiding in drug delivery and release (Cheng et al., 2021). In this review, we discuss current PDT, its basic and clinical research, and the future potential of incorporating nanotechnology into PDT as a lung cancer treatment.

## 2 Photodynamic therapy

### 2.1 History of photodynamic therapy

Oscar Raab discovered that cytotoxicity can be induced using light and a sensitizing agent (acridine dye) in the early 1900s, and his work on PDT was the first to be published (Cengel et al., 2016). When von Tappeiner and Jesionek published their first report on treating patients with basal cell carcinoma successfully with 1% eosin and white light just a few years later, it changed everything (Cengel et al., 2016). Meyer-Betz conducted the first human trial of hematoporphyrin-mediated PDT, which was shown to induce profound and long-lasting generalized photosensitivity. Hematoporphyrin was shown to not only localize to neoplastic tissue but also induce tumor involution when exposed to visible light by capitalizing on its fluorescent properties when illuminated with a Wood’s lamp (365 nm peak emission). PDT using porphyrins and porphyrin derivatives has been studied for its ability to localize in various tumors after intravenous injection for many years (Cengel et al., 2016; Hamblin, 2020). Clinical PDT using the commercially available PS hematoporphyrin derivative (HpD) compound (Photofrin^®^) has been approved for early- and advanced-stage lung cancer in 1993 in various countries. In 1998, it received U.S. FDA approval for early-stage lung cancer. It was expected that with the development of new PSs and their broad-spectrum application for disease, it would gain physician acceptance. Moreover, it was considered that physicians would need to learn how to use PDT to reduce adverse reactions such as light sensitivity and nonspecific localization of the PS in normal tissue. Furthermore, with the development of the diode laser, which reduced the cost of required light sources while also optimizing PDT protocols, it was hoped that physicians would be convinced to use PDT (Dougherty et al., 1998). This however seems to not have been the case where numerous *in vitro*, *in vivo*, and clinical studies have been conducted, yet PDT is still to be recommended for lung cancer treatment.

### 2.2 Research on photodynamic therapy and lung cancer

Since PDT was approved as a clinical treatment for lung cancer, researchers have continued to investigate different PSs and light sources for use in PDT to optimize the treatment. This is because PDT has been shown to have several minor side effects, which were to be overcome by understanding the biological interaction of the PS with its environment and the mechanisms that follow light activation, as well as determining which PSs have optimal photodynamic properties and their localization in cancerous cells.

### 2.3 Photodynamic therapy mechanism

**FIGURE 1:**
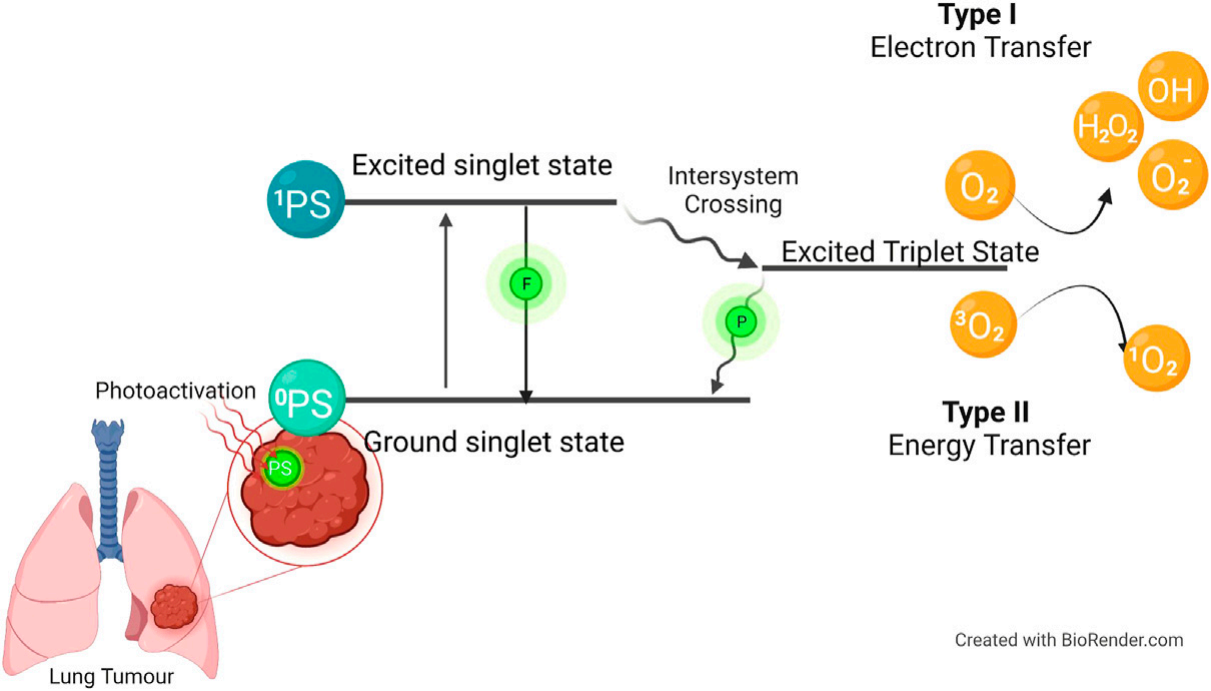
The mechanism of photodynamic therapy (PDT). The photosensitizer (PS) is absorbed when the PS is in its ground state. It goes into its first excited singlet state because of photoactivation. This state can be broken down by emitting fluorescence, or it can cross over to the more stable excited triplet state. Type I is when the PS in its excited triplet state reacts with biomolecules (such as lipids, proteins, and nucleic acids), and the radical mechanism is used to transfer hydrogen atoms. It generates free radicals and radical ions (the type of radical varies depending on the target molecule, such as lipids, proteins, or nucleic acids), which react with oxygen to produce reactive oxygen species. Type II reactions are based on a phenomenon known as triplet–triplet annihilation. In these reactions, the PS in its excited triplet state reacts with oxygen in its triplet ground state. This results in the formation of highly reactive and cytotoxic singlet oxygen.

PDT is a noninvasive form of modern nonionizing radiation therapy. Local or systemic application of a photosensitive compound (PS), which is retained by cancerous cells and tissue through active or passive accumulation, is the basis for this treatment (Castano et al., 2004), combined with single wavelength activation light. The PS molecules absorb the light of the appropriate wavelength, initiating the activation processes of reactive oxygen species (ROS) production leading to the selective destruction of cancer (Castano et al., 2004; Kwiatkowski et al., 2018). Free radicals are formed when the ground state PS is excited into its excited singlet state via photon absorption. The excited singlet state is then converted to a triplet state that interacts with the surrounding molecules via intersystem crossing. When the PS abstracts an electron from a reducing molecule in its vicinity, it produces ROS; when the PS interacts directly with molecular oxygen, it produces singlet oxygen (Benov, 2015). In this two-stage procedure (Figure 1), the harmless PS is activated only via direct illumination, resulting in local tissue destruction, which significantly reduces side effects. Organelles and cell membranes can be damaged directly by PDT components depending on the PS type, concentration, intracellular localization, light fluence, and the location of the radicals. The main benefit of PDT is the treatment site’s high selectivity (Dos Santos et al., 2020; Wang et al., 2021).

**FIGURE 2:**
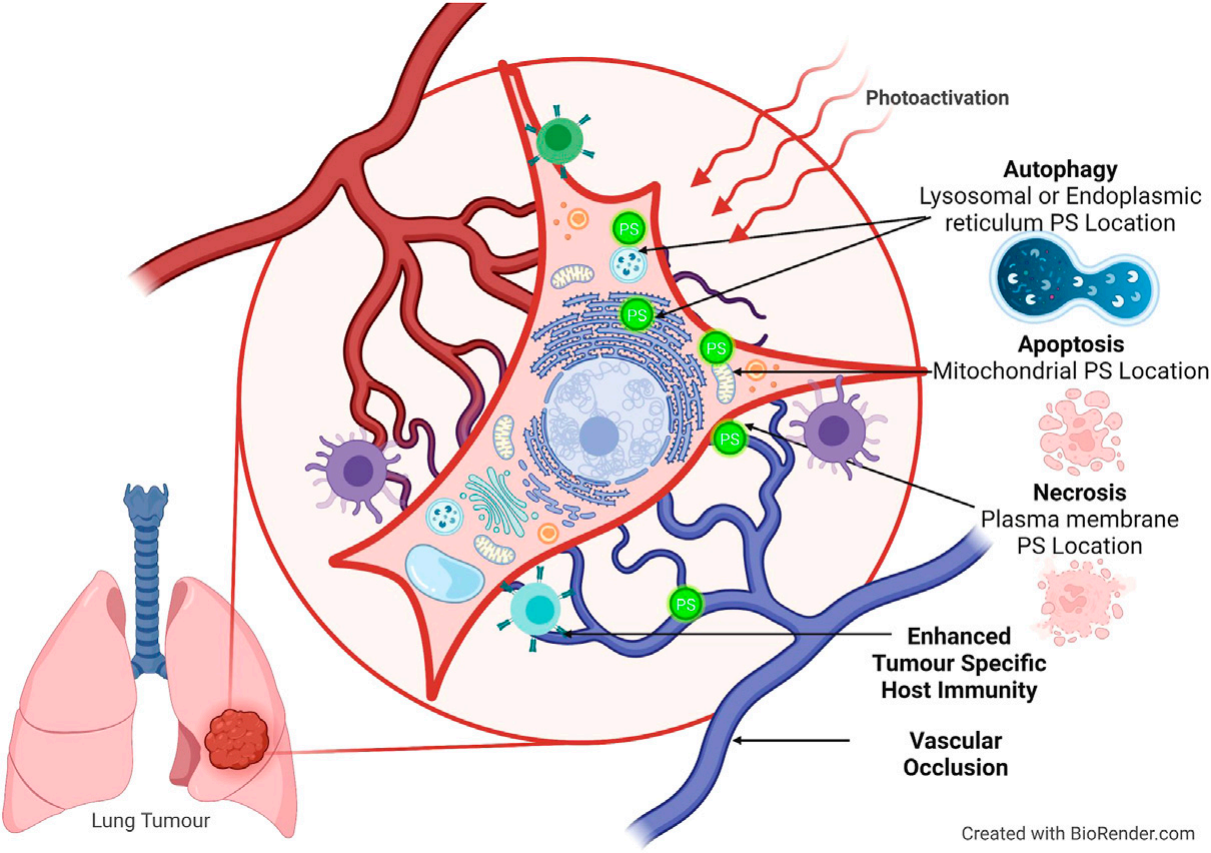
Cell death pathways activated during PDT, according to PS localization. The PS can localize in the cytoplasm and mitochondria and induce apoptosis. Autophagy occurs when there is damage to the lysosomes or endoplasmic reticulum. Necrosis occurs during plasma membrane localization.

The type of organelle damage (Figure 2) and, by extension, the mechanism of cell death are determined via PS localization, according to the findings (Tsubone et al., 2019). The PS can be found in a variety of organelles within the cell, including the mitochondrion, lysosomes, endoplasmic reticulum (ER), plasma membrane, Golgi apparatus, and cytoplasm. Furthermore, clinically, the PS can affect vascular shutdown and activate an immune response. This can lead to various cell death mechanisms including apoptosis, autophagy, and necrosis (Mroz et al., 2011).

Apoptotic cell death occurs when the PS localizes in mitochondria and releases cytochrome C and Bcl-2, where both are associated with direct mitochondrial damage (Oleinick et al., 2002). During cytoplasmic localization, the nuclear factor kappa B (NFB) pathway is damaged, resulting in apoptosis (Castano et al., 2005). This damage to the NFB pathway is significant because it impairs its ability to stimulate antiapoptotic genes (Mroz et al., 2011). When the PS localizes in the lysosomes and ER, it activates the Beclin-1 and the mechanistic target of rapamycin proteins causing an increase in autophagy (Thorburn, 2008). Furthermore, necrosis is visible when the PS causes plasma membrane disintegration (Danial and Korsmeyer, 2004). Indirect cytotoxicity manifested as tumor vasculature breakdown results in microvasculature stasis, causing hypoxia and local nutrient depletion, eventually leading to tumor regression (Wang et al., 2012).

Furthermore, the PDT-damaged cells trigger the immune system that initiates a cascade of chemical signals causing inflammation through activation of leukocyte chemotaxis that produces cytokines. The damaged cells recruit innate inflammatory cells by releasing endogenous signals or danger-associated molecular patterns into the vascular system (Chen et al., 2018). Helper T-cells, cytotoxic T-cells, and regulatory T-lymphocytes are all products of the adaptive immune response in conjunction with innate immunity. A long-lasting antitumor immunity resulting from activation of the adaptive immune system, conversely, serves to control tumor metastasis and aid in the prevention of cancer recurrence following PDT (Nath et al., 2019).

### 2.4 Photosensitizers

The PS is the key component among the three, together with visible light and molecular oxygen, needed to induce photodynamic cytotoxicity. PS properties such as the chemical nature of the PS including molecular weight, lipophilicity, amphiphilicity, ionic charge, and protein binding characteristics can determine its localization (Castano et al., 2004) and effectivity. PS properties that are ideal include strong absorption, with a high absorption coefficient, in the red or near-infrared (NIR) range (650–800 nm), where penetration in tissues is deeper. The energy of the triplet state is greater than 94 kJ/mol, indicating a high quantum yield of triplet state formation (Φ T). The triplet state has a long lifetime (τ T in the long µs range) and a high quantum yield of singlet oxygen formation (Φ Δ). In the absence of light, the PS must not be harmful. The target area is densely loaded with the PS. Excellent biocompatibility, combined with high chemical stability and low photobleaching, enables prolonged photoinduced singlet oxygen yield (Mussini et al., 2022). HpDs were used in the first PS generation. Photofrin^®^ (porfimer sodium) was first approved for clinical use in 1993 and has since been used to treat a variety of cancers, despite its drawbacks of low chemical purity; prolonged half-life; excessive accumulation in normal cells and the skin, which can result in photosensitivity; and a low tissue penetration wavelength of 630 nm (Agostinis et al., 2011). Despite its disadvantages, Photofrin is still widely used for PDT cancer treatment including lung cancer. The disadvantages of first-generation PSs prompted a concerted effort to develop novel photosensitizing agents or second-generation PSs with a well-defined chemical identity, enhanced photophysical properties, and increased tumor selectivity.

PSs from the second generation are more effective and technically superior to those from the first generation. They have been modified to achieve a higher level of chemical purity and a higher quantum yield for singlet oxygen formation. Second-generation PSs with modified cores were designed for mitochondrial-specific targeting. Second-generation PSs are excited at a longer wavelength in the visible or NIR region of the spectrum; thus, deeper light penetration improves treatment efficacy. The majority of second-generation PSs are porphyrin- and chlorin-based (Baskaran et al., 2018). There are a variety of ways to make second-generation PSs, including macrocycle or substituent modification, or different molecular structures (Josefsen and Boyle, 2008). Chlorins, metalloporphyrins, phtalocyanines, cyanines, phenothiazines, verteporphin, pheophorbides, porphycenes, protoporhyrin IX precursor, dipyrromethenes, hypericin, purpurins, and xanthenes are examples of second-generation PSs that have been developed over the years. Even though multiple photosensitive molecules have been explored and devised, only a limited number of PSs have been approved for clinical use (Table 1) (Baskaran et al., 2018; Zhang et al., 2018; Mussini et al., 2022). Up to date, only two PSs are approved for lung cancer, namely, Photofrin^®^, which has been approved worldwide, and Laserphyrin^®^ (Talaporfin), a mono-L-aspartyl chlorin that was approved in Japan in 2004.

**TABLE 1:** Clinically approved photosensitizers.

Photosensitizer	Cancer application	Country	Chemical base structure	Activation wavelength (nm)
Photofrin^®^	**Lung**, gastric, bladder, cervical, and esophageal	Canada, Japan, the United States, and Europe	Hematoporphyrin	630
Foscan^®^	Head and neck	European Union, Norway, and Iceland	Chlorin	652
Metvix^®^	Nonhyperkeratotic actinic keratosis and basal cell carcinoma	United Kingdom, EMEA, the United States, and Canada	Protoporhyrin IX precursor	570–670
Levulan^®^	Actinic keratosis, HPV	EMEA, the United States, Austria, and China	Protoporhyrin IX precursor	635
Visudyne^®^ verteporfin	Age-related macular degeneration, basal cell carcinoma	Switzerland, China, and the United States	Benzoporphyrin	690
Laserphyrin^®^ talaporfin	Early centrally located **lung** cancer and glioma	Japan	Chlorin	664
Redaporfin^®^	Biliary tract cancer	Portugal	Bacteriochlorin	749
Tookad^®^	Prostate	Europe, Israel, and Mexico	Bacteriochlorophyll	762

PSs can be given intravenously and topically in clinical and preclinical settings. For superficial lesions, topical administration is advised, whereas oral administration is advised when topical delivery is inaccessible to the tumors (Foster et al., 2010). The drug–light interval varies depending on the PS used, averaging between 6 and 48 h. After sufficient time has passed for absorption, a specific wavelength of light is briefly shone on the affected area. The light dose is determined by the fluence rate, the PS’s molar extinction coefficient, and the concentration of the PS in the tissue. Light is administered using a nonthermal diode laser, arc lamp, fluorescent light, or LEDs (Breskey et al., 2013; Van Straten et al., 2017). For lung cancer PDT, the PS is administered intravenously (Shafirstein et al., 2016) and used as an endobronchial therapy (Simone et al., 2012).

One of the major challenges in cancer therapy is the effective and safe delivery of anticancer agents. The majority of anticancer drugs are toxic to normal cells, have low bioavailability, and are unstable *in vivo* (Amreddy et al., 2018). With approved PSs still having phototoxicity issues, PS development has centered on developing photosensitive drugs with ideal photonic and biological properties. There have been several experimental and preclinical studies evaluating the effectiveness, safety, and toxicity of PSs (Gomes et al., 2018). Studies have found that the PSs used had low *in vivo* toxicity and effective tumor regression (Fong et al., 2015). Results of *in vivo* studies for lung cancer using Lewis lung carcinoma (LLC) mouse models showed that photofrin-based PDT appears to cause a natural innate immune response used to maintain homeostasis following PDT-induced acute tumor injury, indicating that PDT used to treat solid tumors provides an overall benefit in terms of long-term tumor control (Cecic et al., 2005; Wachowska et al., 2014). The PS 5-aminolaevulinic acid (5-ALA) inhibits vascularization in lung metastases, resulting in significant inhibition of primary tumor growth and suppression of metastatic processes (Lisnjak et al., 2005). A bacteriochlorin study found that using PDT against LLC tumors increases the average lifespan of the animals. Treatment focused on vascular destruction (V-PDT) results in a highly effective long-term antineoplastic response mediated by a strong deprivation of blood supply. Tumors in 67% of LLC-bearing mice treated with V-PDT completely regressed and did not reappear for over a year (Karwicka et al., 2019).

### 2.5 Lung cancer photodynamic therapy in the clinic

One of the first endoscopic PDT procedures for human lung cancer was performed in 1980 on 13 lung cancer cases and in one case of severely atypical squamous metaplasia. One patient had complete regression of the tumor mass and remained disease-free 16 months after the treatment. Another with severely atypical squamous metaplasia had a full recovery (Hayata et al., 1982). The second PDT case involved a 59-year-old woman with early-stage squamous cell carcinoma with inoperable cancer due to poor cardiopulmonary function. The lesion disappeared within a week, and the patient stayed disease-free for more than 5 years (Kato et al., 1986). Over the years, clinical trials have shown that PDT prolonged survival and palliation in inoperable, partially or totally obstructing NSCLC (Diaz-Jimenez et al., 1999), effective in palliation of advanced-stage SC/NSCLC (Moghissi et al., 1999), had no morbidity or mortality, and improved symptoms and quality of life when used in conjunction with chemotherapy in advanced NSCLC where curative operations were contraindicated (Kimura et al., 2015). The combined high dose rate brachytherapy and PDT for various stages (I–IV) of endobronchial tumors showed to be well tolerated and can achieve prolonged local control with acceptable morbidity (Weinberg et al., 2010). After treatment failure with surgery, radiotherapy, and chemotherapy, stage II–IV intractable bronchial lung cancer treated using photofrin had a total response rate CR + PR of 86.7%, and the mean tumor obstruction percentage also decreased (Cai et al., 2013). The use of the second-generation Radachlorin®–based PDT alone on advanced NSCLC showed treatment efficacy and safety, with a 1 year survival rate of 70% posttreatment (Ji et al., 2013). PDT using 2-[1-hexyloxyethyl]-2-devinyl pyropheophorbide-a showed to be safe and effective against NSCLC with a CR of 72.7% at 6 months (Dhillon et al., 2016).

### 2.6 Limitations of photodynamic therapy

Aside from being less invasive than traditional surgical resection, PDT also has several advantages over other forms of lung cancer treatment including greater target specificity, less harm to healthy tissues nearby, and negligible side effects on the body as a whole. Administering PDT is simple to perform, can be done in an outpatient setting, and does not leave any scars after healing. Chemotherapy and radiotherapy are far more toxic than photosensitive agents and visible light sources. Radiation, chemotherapy, and photothermal therapy can all be used in conjunction with PDT. PDT, like other treatments for lung cancer, has its drawbacks.

The relative importance of PDT is greatly influenced by various factors, including the type and dose of PS used, the amount of time between the administration of the PS and exposure to light, the total light dose and the fluence rate, the concentration of oxygen in the tumor, and perhaps other factors that are still poorly understood (Agostinis et al., 2011).

The double-edged sword of PDT for lung cancer treatment is that tumor cell destruction on both parenchyma and stroma has the potential to be hampered by the reliance on ROS generation in the presence of oxygen because not all tumor tissue is properly vascularized, and it can result in oxygen and PS deficiency (Van Straten et al., 2017). Nonspecific uptake of PS into normal cells, conversely, can have negative consequences. When PS is localized in the lung vasculature and tissue as a result of PDT treatment, it can have a similar effect on normal lung cells, destroying healthy tissue and causing unwanted side effects (Van Straten et al., 2017). This is because the lung is a highly vascularized tissue. Because the lungs’ primary function is respiration, where gas exchange is performed by distal lung capillaries, microvasculature is a key component of lung tissue by nature. These capillaries wrap around the internal alveolar wall, allowing for the exchange of nutrients, hormones, and ions. Endothelial cells control vascular contraction, enzymes, immune function, and platelet adhesion in lung capillaries, which are supported by pericytes and fibroblasts. The arterioles, which are made up of endothelial and smooth muscle cells and receive blood from the medium and large pulmonary arteries, which are made up of elastic laminae, control blood flow volume to the capillaries (Tsuchiya et al., 2020). However, the concept of vascular targeted PDT, which involves highly vascularized tumors that result in an increased PDT response because the PS distribution allows for effective oxidant formation and endothelial and subendothelial cell destruction, has been shown to improve therapeutic efficacy. Endothelial PDT damage induced by ROS causes cell rounding, widening the interendothelial junctions exposing underlining tissue, and may also release clotting factors where platelet aggregation leads to thrombus formation, vascular occlusion, and vasoconstriction. The impaired blood flow and blood vessel destruction, in time, will result in tissue hypoxia, nutrient deprivation, and tumor destruction (Krammer, 2001).

Temporary side effects of PDT include photosensitivity, which can last for months. It is usually mild to moderate in severity and does not require treatment. Systemic immune response manifested as localized swelling at the injection site is frequently seen following PDT treatment. Inflammation in the short term serves a protective purpose. The innate immune system oversees orchestrating the inflammation induced by PDT. Changes in tumor vasculature are an early indicator of PDT-induced inflammation (Hwang et al., 2018).

It has been noted that PDT is limited in treating large tumor masses and has a treatment depth limit. Only superficial lesions can benefit from PDT because visible light only penetrates 5–10 mm into tissues. To get around these limitations, new fiber optic and microendoscopic technologies have been developed, allowing for the precise placement of fibers inside the tumor site using interstitial, endoscopic, intraoperative, or laparoscopic light distribution devices (dos Santos et al., 2019).

PS and light doses have been established in experimental research, but no clinical agreement on how PS and light should be measured has been reached, nor does there exist a widely accepted definition of the dosage. It is unfeasible to achieve the highest response rates using standardized protocols because the optimal PS and light doses, as well as the drug–light time interval, may differ from case to case. Therefore, improving dosimetry has been an ongoing goal for clinical PDT usage. A PDT dose dosimeter was developed by Kim and colleagues for use in pleural PDT with Photofrin in their study. They used an isotropic fluorescence detector to simultaneously measure both the light fluence and the PS concentration in the same treatment location (Kim et al., 2016). This method of dosimetry measurement may be a viable option for future studies.

## 3 Nanophotodynamic therapy as a novel approach to photodynamic therapy for lung cancer treatments

The development of nanotechnology has accelerated significantly over the last decade. Combining PSs and nanomaterials has the potential to increase the efficacy of PDT while minimizing its side effects. The use of nanoparticles enables the development of a targeted method that is focused on specific receptors, thereby increasing PDT selectivity (Kwiatkowski et al., 2018) and sensitivity (Xiang et al., 2021).

Nanotechnology is a fusion of chemistry, biology, applied physics, optics, digital analysis, and materials science. Designers of nanoscale structures can use this rapidly expanding multidisciplinary field to create and manipulate nanoscale structures (Bayda et al., 2019). These structures are known as nanoparticles, and their increasing popularity and publication show their increasing interest. These properties, such as rigidity, hydrophobicity, size, and charge, show how effective this technology is for healthcare or treatment issues. Nanomedicine is a specialized application of nanotechnology that allows for accurate diagnosis and therapy. One such example is nanoparticle drug delivery, which is reportedly undergoing substantial research in the fields of molecular nanotechnology and nanovaccinology. Nanomaterials can deliver hydrophobic-like treatment to cancerous regions by circumventing biological barriers. Nanoparticles of gold, silver, and platinum are particularly attractive for such applications. Localized surface plasmon resonance is caused by their optical absorption and scattering properties. They can interact with biomolecules both on the cell membrane and within. These nanoparticles have the potential to be used to diagnose and treat serious illnesses such as cancer (Mfouo Tynga and Abrahamse, 2018).

**FIGURE 3:**
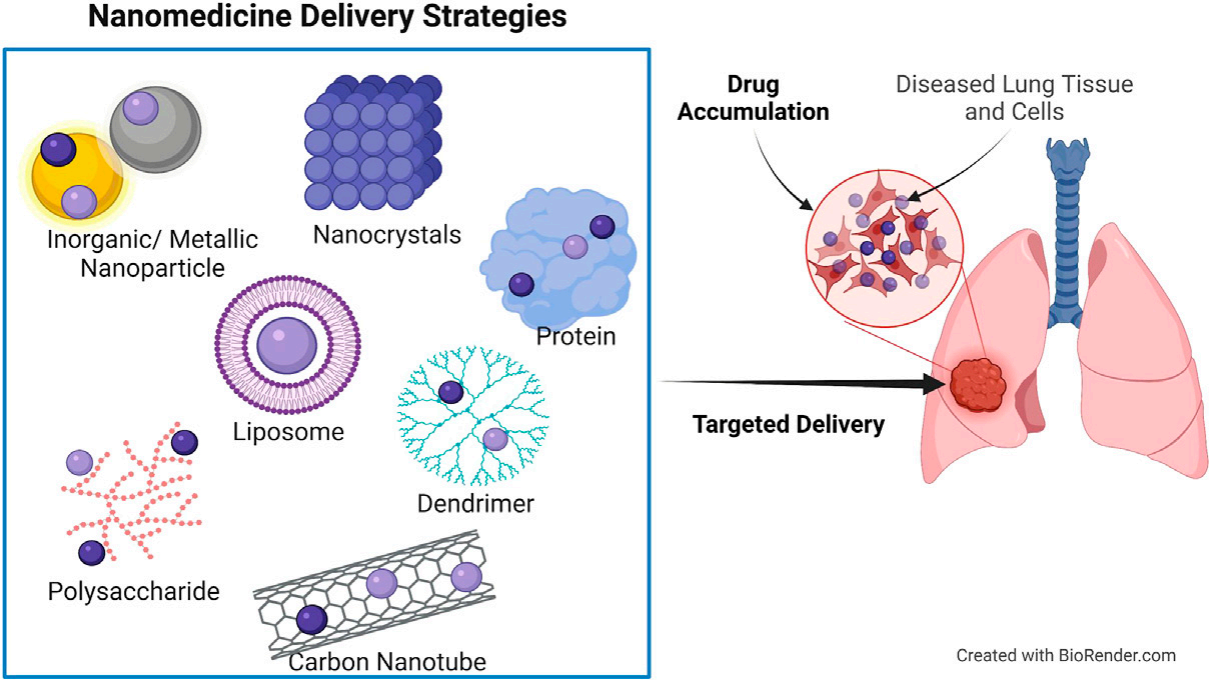
Nanodrug delivery systems used for site-targeted distribution and improved bioavailability. Protein and polysaccharide nanoparticles, liposomes, dendrimers, inorganic/metallic nanoparticles, nanocrystals, and carbon nanotubes are all examples of nanoparticles used in nanomedicine.

Nanoparticles used in clinical applications are multifunctional, with the most common being drug delivery. It is possible to better target the tumor and maximize the effectiveness of anticancer drugs by utilizing nanocarriers during cancer therapy. Using nanoparticles in drug delivery is justified by the fact that conventional therapeutic agents are unable to effectively target tumor tissue and treat the disease (ud Din et al., 2017). Nanodrug delivery systems currently use polymers, micelles, dendrimers, proteins, liposomes, and metallic nanospheres or tubes as carriers (Figure 3) (Khodabandehloo et al., 2016).

Nanoparticles can be delivered either passively or actively, depending on the application (Egusquiaguirre et al., 2012). Tumor microenvironment features such as enhanced permeability and retention (EPR) and acidic conditions are at the root of passive nanoparticle drug delivery. Enhanced metabolic capacity and increased neovascularization, which are frequently porous with gap junctions between endothelial cells, result in the EPR effect. Passive targeting is possible because of these breaches, and nanocarrier systems accumulate selectively in tumor cells. Extracellular tumor environments become acidic because of the glycolytic pathway being activated to meet the high energy demands of rapidly proliferating cells. Nanocarrier systems such as liposomes dissolve in tumor cells’ acidic environments, releasing therapeutic agents. Passive targeting has limitations because of the mucosal barrier and nonspecific drug delivery. Active routes aim to improve selective targeting while circumventing these limitations. Conjugation of active nanocarrier systems to biomolecules such as ligands and antibodies enhances their ability to target tumor cells with high specificity (Mfouo Tynga and Abrahamse, 2018).

Therapeutic outcomes are improved by delivering the drug’s active form at the lowest possible dose and with the least possible activity loss and side effects when using targeted anticancer agents. Two direct advantages of targeted anticancer therapy are the prolongation of drug accumulation in cancer cells and the resulting improvement in the therapeutic index. Bioconjugation and the bonds formed between carriers and drugs, as well as between carrier–drug systems and cancer cells, are therefore crucial. Bioconjugation, noncovalent and covalent interactions, is used in the construction of an effective anticancer drug delivery system (Werengowska-Ciećwierz et al., 2015). Amide bonds are formed on the surface of nanocarriers by a stable chemical reaction. To achieve minimal activity loss, high complex stability, dispersion, prolonged biodistribution, and high accumulation in cancer cells (Ishida et al., 2001), as well as improved therapeutic outcomes, modified targeted delivery systems can be used (Zeng et al., 2006). When thioester bonds are formed between nanocarriers and ligands, the delivery systems’ selectivity and biodistribution lengthen. Chemical reactions, such as molecular rearrangement and the formation of disulfide bonds, are necessary for this formation. Ligands and nanocarriers may form disulfide bonds during the conjugation process, increasing the affinity of these delivery systems for cancer cells (Nobs et al., 2004). Hydrazide and acetyl groups on ligands form acetyl–hydrazine bonds with nanocarriers. It can now survive in blood and an immunologically hostile environment because of these modifications, which give the entire system more control and stability (Nobs et al., 2004). In addition, bicyclic products can be generated using the Diels–Alder reaction to increase the affinity of cancer cells for ligands (Liu et al., 2007). Using the same reaction, ligands can be attached to nanocarriers (Shi et al., 2007). By contrast, noncovalent interactions are poor connections with delivery systems that can be easily severed during therapy. Quality and safety are being jeopardized because of the potential for side effects from alterations to the delivery system. Click chemistry, a class of biocompatible small molecule reactions commonly used in bioconjugation, has been used to modify biological ligands after the nanoparticles have been synthesized without changing their function. Click chemistry reactions are orthogonal with other functional groups, have a favorable reaction rate in aqueous conditions, and generate minimal byproducts (Xiang et al., 2021). This may provide purification options for these nanoconjugate systems prior to their use in targeted cancer therapy, which are frequently overlooked after the assembly processes (Mfouo Tynga and Abrahamse, 2018). Furthermore, to determine whether the conjugation bonds between the nanoparticle and PS have no detrimental effect on the PS molar extinction coefficient or optical properties, characterization of the conjugate is always necessary before treatment.

We reviewed recent updates on the use of nanoPDT for lung cancer, where studies used PSs that were loaded onto nanoparticles and photoactivated. An *in vitro* study using zinc phthalocyanine loaded onto poly-ɛ-caprolactone nanoparticles showed that upon photoactivation using 660 nm red light, A549 lung cancer cell viability decreased following time-dependent phototoxicity with an increase in light dose (da Volta Soares et al., 2011). Another study explored the effects of using a nanobubble-encapsulated hybrid nanosystem that can be monitored via US and fluorescent imaging and activated by NIR light. The researchers used a hybrid nanosystem consisting of upconversion nanoparticles (UCNPs) and mesoporous silica-coated gold nanorods (AuNR@mS) with merocyanine 540 PS to realize dual phototherapy. Characterization of the nanosystem indicated the enhanced luminous intensity of the nanoparticle by holmium ion and emitted green and red light and showed excellent stability. *In vitro* A549 cells were confirmed to undergo apoptosis because of mitochondrial ROS destruction using the AuNR@UCNP@NB with US releasing the nanocomposite and 808 nm laser activation. Furthermore, *in vivo* experiments, where A549 lung cancer cells were subcutaneously injected into the right thigh of the mouse and allowed to grow into a tumor of 21 mm^3^, showed dual model imaging and tumor suppression photodynamic effects (Huang et al., 2020). Researchers assessed the effectivity of curcumin when used in a nanoPDT system. Curcumin was encapsulated using solid lipid nanoparticles using an activation wavelength of 430-nm light–emitting diode and studied on NSCLC A549. Results revealed increased cytotoxicity, efficient drug delivery into the mitochondrion, and increased stability of the PS and ROS production, with apoptosis as the cell death mechanism (Jiang et al., 2017). Rossi et al. explored the use of X-ray excited PDT using porphyrin conjugated silicone nanowires *in vitro*. A549 lung adenocarcinoma cells were treated using tetra (N-propynyl-4-aminocarbonylphenyl) porphyrin (H2TPACPP) loaded onto SiC/SiOx nanowires. Results showed good spectral properties of the nanoparticle, singlet oxygen formation, and reduced clonogenic formation of the cells’ post-X-ray activation using a dose of 2 Gy (Rossi et al., 2015). Likewise, Yang and colleagues used low-dose X-ray photoactivation to induce a photodynamic reaction in A549 cancer cells *in vitro* and *in vivo* treated with cerium-doped calcium carbonate nanoparticles. The study found that nanoPDT induced cancer cell cytotoxicity and significant ROS production, and it induced tumor ablation with no harm to normal tissue, indicating effective delivery and localization (Yang et al., 2019b). In another upconversion nanoparticle-based study, researchers loaded camptothecin a chemo drug, Chlorin e6, and carboxyl-mPEG onto UCNPs. Upon laser activation of 980 nm, the conjugate emitted a narrow emission band of 645–675 nm absorbed by the Ce6. Results indicated complete elimination of NCL-H460 lung cancer *in vivo*, along with cancer-targeted fluorescent imaging and dual chemo-PDT ROS activation (Yue et al., 2016). In another study using X-ray luminescence, a conjugate consisting of a scintillator core (LiGa5O8: Cr), whose emission matched the excitation wavelength (720 nm) of the PS 2,3-naphthalocyanine, was encapsulated into mesoporous silica nanoparticles. The nanoparticles (NC-LGO:Cr@mSiO_2_) were then conjugated to cetuximab and systematically administered. Findings show that the conjugate penetrates deep-seated lung tumors (H1299), has good optical properties for bioimaging, and leads to tumor suppression upon activation with X-ray at 4 Gy with no adjacent tissue toxicity (Chen et al., 2017). There are still a lot of hurdles to overcome before these PDT nanoplatforms are ready for use in the clinic.

Despite significant advancements over the last few years in cancer management, treatment effectiveness remains stagnant. Treatment resistance, metastatic spread, and tumor recurrence could all be caused by a small subpopulation of cancer stem cells (CSCs) with special abilities. This means that cancer treatment is severely hindered by the presence of CSCs, and more efforts must be made to develop cancer therapies that simultaneously target CSCs (Marzagalli et al., 2021). Anti-CSC treatments can be made more effective and sensitive by using nanomedicines. This is a viable and promising approach. Nanoparticles could be used to better deliver anticancer agents while also targeting CSCs, which could reduce the role of CSCs in self-renewal, proliferation, tumor progression, drug resistance, recurrence, and metastasis in many neoplastic conditions (Lu et al., 2016). Additional benefits of nanomaterials include improved stability and bioavailability of anti-CSC agents as well as a reduced risk of side effects for normal stem cells. Achieving greater therapeutic efficacy against drug-resistant cancers with free drugs is difficult, but nanodelivery systems can increase effectiveness. Nanocarriers affect the self-renewal and differentiation of CSCs, their proliferation, and the regulation of metabolic activities in drug-resistant cells and drug efflux transporters. It is probable to concurrently kill cancer cells and eliminate drug-resistant CSCs with the use of nanocarriers in therapy (Mfouo Tynga and Abrahamse, 2018).

In a more recent study using nanoPDT on A549 lung CSCs, researchers used a gold nanoparticle delivery vehicle to deliver AlPcS_4_Cl PS, along with conjugating it to the CSC marker CD133. Results indicated that the conjugates localized in the perinuclear region and upon photoactivation using a 660 nm LED induced apoptotic cell death to the point of eradication, compared with the PS alone, which had killed 50% of the CSCs (Crous and Abrahamse, 2020).

## 4 Discussion

Early detection, appropriate treatment, and cancer patient care can help to reduce the cancer burden. When detected and treated early, many cancers have a high chance of being cured. Current lung cancer treatment options have been shown to have negative side effects, which can result in treatment resistance and metastasis. PDT had previously demonstrated the ability to treat patients with minimally invasive lung cancer, particularly those with early-stage central lung cancer. PDT can be used alone or in combination with other treatments for lung cancer if the disease is incurable and other options have failed or been refused. PDT is, however, underutilized in clinics for the treatment of lung cancer and other tumors.

PDT was first approved clinically for the treatment of certain tumors over 25 years ago. Researchers have been studying the mechanism by which PDT exerts antitumor activity for decades. The first PDT mechanism was identified by observing significant variation in the level of antioxidant molecules expressed in cancer cells. PDT’s primary advantage is that it is a highly selective method of destroying undesirable cells and tissues. PSs are molecules that are covalently linked to other molecules that have an affinity for cancer or specific tumor receptors. Most PSs used in cancer therapy have a tetrapyrrole structure, like the protoporphyrin found in hemoglobin. Combinations of distinct therapeutic modalities with nonoverlapping toxicities are a frequently used strategy in modern oncology for increasing treatment efficacy. Neoadjuvant therapy is frequently used to shrink tumors and improve the chance of a successful surgical procedure. According to some studies, postoperative PDT improved mean survival time when compared with standard postoperative care alone. PDT established itself as a viable alternative to palliative chemotherapy or radiotherapy in patients with unresectable lung cancer, achieving an overall response rate of nearly 87% and significantly improving patients’ quality of life. Sensitization of tumor cells to PDT and disruption of PDT-induced cytoprotective molecular responses in surviving tumor cells improve antitumor efficacy. Not only does PDT kill the targeted cells and cause damage to the tumor-associated vasculature, but it also stimulates an antitumor immune response. According to some studies, PDT induces an immune response that can control both localized and metastatic cancer. The beneficial effect of intraoperative PDT on mesothelioma patients may be due to immune response modulation (Simone and Cengel, 2014). Yang and colleagues created a system consisting of dual-modal single–walled carbon nanohorns that stimulates systemic immune responses against tumor metastasis and relapse in patients with advanced metastatic cancer (Yang et al., 2020). As a result, developing a new PDT and immune checkpoint blockade therapy combination for metastatic lung cancer could be beneficial. With PDT, there is less long-term morbidity, and it does not preclude future treatment options for patients with recurrent disease compared with surgery, chemotherapy, or radiotherapy. These studies demonstrate that PDT can be easily integrated into routine lung cancer care regimens, palliatively to improve therapy outcomes, or as a standalone treatment (Wang et al., 2021).

Despite significant advances in lung cancer treatment with PDT, there are still some limitations and minor side effects that could be improved, including drug specificity and localization, as well as light delivery methods. PSs conjugated to a specific nanoparticle platform could be developed to increase penetration. In addition, nanoplatforms equipped with specific receptor–based detectors, such as antibody constructs, monoclonal antibodies, or small molecule inhibitors, may aid in PS delivery to lung cancer cells (Mokwena et al., 2018). Nanomedicine-assisted cancer therapy is more specific for cancer cells, has fewer side effects, and is effective *in vitro* and *in vivo*. In comparison with free drugs, nanocarriers used in drug delivery systems are extremely capable of penetrating the CSC niche, killing cancer cells while also eradicating drug-resistant CSCs, resulting in up to 100-fold therapeutic efficacy against drug-resistant cancer. When using nanotechnology for therapy, precautions should be taken because of the unknown effects of prolonged exposure to biological environments (Mfouo Tynga and Abrahamse, 2018). If all the disadvantages and clarifications are addressed, nanomedicine in combination with other enhanced therapies would be a more viable option for cancer eradication. Nanomedicine’s dominance over current treatment options in cancer therapy is likely to continue, potentially resulting in the effective eradication of drug-resistant cancers. Medical applications based on nanoparticles have already demonstrated significant benefits in the treatment of a variety of diseases. This novel technology has the potential to address long-standing problems associated with cancer recurrence and drug resistance in cancer therapy (Mfouo Tynga and Abrahamse, 2018). The chemical alteration, nanodelivery systems, or antibody conjugation of third-generation PSs are being investigated thoroughly *in vitro*, and more *in vivo* experiments are necessary for the validation of its effectivity to be included in clinical trials and clinical development. Lung cancer patients can benefit from the use of novel PDT systems, nanoparticle-based PSs, and improved imaging and surveillance systems. More research is required into the use of nanoPDT for lung cancer treatments.

## Data Availability

Any data supporting this review are from previously reported studies and datasets, which have been published. These prior studies are cited at relevant places within the text as references.
